# Common Complications in Aesthetic Breast Augmentation

**Published:** 2015-08-21

**Authors:** Sarah Chang, Arvind Gowda, Vasilios Mavrophilipos, Nina Semsarzadeh, Devinder P. Singh

**Affiliations:** Division of Plastic Surgery, University of Maryland School of Medicine, Baltimore

**Keywords:** breast augmentation, capsular contracture, double-bubble deformity, symmastia, neosubpectoral pocket

**Figure:**
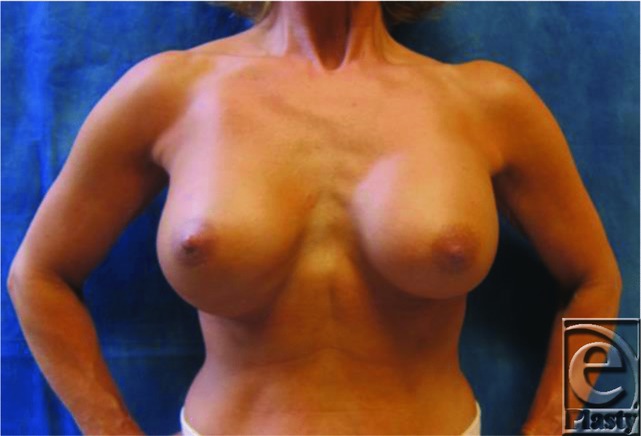


## DESCRIPTION

A 48-year-old woman, 34 weeks status post primary cosmetic bilateral breast augmentation, presented with right double-bubble deformity. The patient reported persistent right breast pain, particularly on contraction of the right pectoralis muscle.

## QUESTIONS

1. **What is capsular contracture?**
2. **What is double-bubble deformity?**
3. **What is symmastia?**
4. **What is neosubpectoral pocket?**

## DISCUSSION

Capsular contracture is a common complication associated with breast augmentation. One hypothesis regarding the etiology and pathogenesis suggests that following the surgical procedure and placement of the implants, the body initiates a foreign body response that leads to the formation of a collagenous capsule surrounding the implant.^1^ In the setting of capsular contracture, this response consists of an inflammatory reaction, fibroblast recruitment, collagen fiber formation, and myofibroblast formation. Ultimately, this process results in a firm, and sometimes painful, capsule. Certain factors including subclinical infection, biofilm formation, bleeding, incision location, radiation therapy, and implant compromise may influence the likelihood of a patient developing a capsular contracture.

The double-bubble deformity is another common complication of breast augmentation surgery. Patients characteristically present with 2 parallel, transverse, and curved folds across their inferior breasts formed by the native breast tissue and the prosthetic implant. While its etiology is still debated, major theories include spontaneous upward (type I) or downward implant migration (type II), capsular contracture, postpartum atrophy, and glandular ptosis.^2^ Predisposing anatomic risk factors include tuberous breasts, constricted inframammary folds, and a short nipple-to-inframammary fold distance.^3^ The deformity can be corrected in a variety of ways including capsulorrhaphy, a “neosubpectoral pocket,” and conversion from subpectoral to submammary plane and vice versa.^3^

Symmastia after breast augmentation is a relatively rare complication and may have an early or delayed presentation. The complication arises when constant pressure or excessive dissection of the patient's midline causes a continuous capsular pocket to form instead of distinct bilateral pockets. This process results in a medial displacement of one or both of the implants beyond the midline, confluence of the breasts, and a loss of skin adherence to the sternum.^4^ Patients are more likely to develop symmastia if implants are inappropriately sized or placed too close to the midline or if the patient has existing chest wall deformities.^5^ Common correction techniques include medial capsulorrhaphy, where the capsule is sutured and reattached to the sternum, or the “neosubpectoral” pocket technique.

The “neosubpectoral” pocket is a versatile technique in secondary breast augmentation that can be used to treat both double-bubble deformity and symmastia.^5^ Rather than repair the existing capsular pockets, the technique creates a new pocket between the pectoralis major muscle and the anterior capsule surrounding the implant.^5^ Once the new pocket is developed, the original pocket is closed using a capsulorrhaphy to prevent communication with the original pocket. Several groups have reported on the durability and technical feasibility of this technique.^5^

Breast augmentation surgery using implants is a very common procedure that may lead to various complications. These complications may include but are not limited to a capsular contracture, “double-bubble” deformity, and symmastia. Both implant- and technique-related factors, such as the size of the implant or excessive dissection during the surgery, may influence whether an individual patient will experience complications. Common corrective treatments include resizing and reapproximating the capsule pocket through capsulorrhaphy, changing the positioning or size of the implant from subpectoral to submammary or vice versa, or creating a new pocket between the pectoralis muscle and capsule, known as the “neosubpectoral” pocket.
